# Flow-cytometric analysis in traumatic brain injury to evaluate immunosuppression

**DOI:** 10.1186/cc13417

**Published:** 2014-03-17

**Authors:** S Di Valvasone, L Perretta, M Bonizzoli, F Liotta, F Annunziato, F Socci, P Ruggiano, A Peris

**Affiliations:** 1 Careggi Hospital, Firenze, Italy

## Introduction

Flow-cytometric analysis is still restricted to cancer and immunocompromised patients. There are no clinical studies that evaluate the immunological changes in traumatic brain injury (TBI) patients. The objective of this study is to determine whether patients with severe TBI (GCS <9) manifest early (<48 hours after injury) signs of immunosuppression and whether this condition increases the incidence of infection during the ICU stay.

## Methods

We retrospectively analyzed data from 54 patients, including 10 patients with isolated brain injury and 44 patients with brain and extracranial injuries. The flow-cytometric analysis was performed within 48 hours of trauma. Collected data are shown in Table 1.

**Table 1:** Collected data

Age (years), mean ± SD	55.43 ± 23.12
Sex (M/F)	44/10
Body mass index, mean ± SD	25.51 ± 2.69
ISS, mean ± SD	29.04 ± 26.57
RTS, mean ± SD	5.76 ± 1.53
TRISS, mean ± SD	68.73 ± 28.77
Admission SAPS II, mean ± SD	44.96 ± 14.87
Admission GCS, mean ± SD	7.63 ± 3.88
Flow-cytometric analysis day (days), mean ± SD	1.69 ± 3.34
Discharge GCS, mean ± SD	10.48 ± 3.19
ICU LOS, mean ± SD	10.67 ± 8.08

## Results

Preliminary analysis is limited to descriptive statistics that show an immediate immunosuppression condition after TBI, as established by reduction of CD4^+ ^T lymphocytes and CD8^+ ^T lymphocytes. Significant data are collected in Table 2.

**Table 2:** Flow-cytometric data

		Normal value
WBC (×10^9^/l)	10.67 ± 4.82	4 to 10
Total lymphocytes (×10^6^/l)	1,123.15 ± 547.02	1,200 to 3,000
CD3^+ ^lymphocytes (×10^6^/l)	67.11 ± 11.86	1,100 to 1,700
CD3^+^CD4^+ ^lymphocytes (×10^6^/l)	493.07 ± 313.92	600 to 1,400
CD3^+^CD8^+ ^lymphocytes (×10^6^/l)	250.65 ± 171.94	300 to 900
CD4^+^DRM^+ ^lymphocytes (×10^6^/l)	23.19 ± 15.09	
CD8^+^DR^+ ^lymphocytes (×10^6^/l)	16.26 ± 20.83	

## Conclusion

In severe TBI patients, an immunosuppression state is early developed. It is relevant to establish whether this condition could affect the course and prognosis of ICU patients.

